# Revaluation of Granadilla (*Passiflora ligularis*): Analysis of Phenolic Compounds and Their Antioxidant Activity

**DOI:** 10.1155/ijfo/7032985

**Published:** 2026-08-10

**Authors:** Fabiola N. Cuyo-Jallasi, Harry R. Yucra-Condori, Miriam D. Hubinger, Julio C. Rodríguez-Díaz

**Affiliations:** ^1^ Academic Department of Food Industry Engineering, National University of San Agustín of Arequipa, Arequipa, Peru; ^2^ Faculty of Food Engineering, University of Campinas, Campinas, Brazil, unicamp.br

**Keywords:** antioxidant capacity, extraction, *Passiflora ligularis*, phenolic compounds, ultrasound

## Abstract

This study is aimed at evaluating the effect of solvents such as ethanol and methanol on the extraction of phenolic compounds with antioxidant capacity from granadilla (*Passiflora ligularis*). Extraction was assisted with ultrasound (40 kHz, 30°C). The sample‐to‐solvent ratio was 1:14, and extraction was performed separately for seeds and peels. The proximate composition of peel and pulp stood out for their high carbohydrate content (52.12 and 90.99 g/100 g d.m., respectively), whereas the seeds showed values of fiber, protein, and fat of 29.48, 26.33, and 23.77 g/100 g d.m., respectively. Methanol was the most effective solvent for the extraction of phenolic compounds, and seeds were the highest source of these compounds, reaching a value of 36.26 mg GAE/g extract. The in vitro antioxidant capacity had similar behavior, with values of 7816.9 *μ*mol Fe (II)/g d.m. extract for the FRAP assay and 1161.507 mmol TE/100 g extract for ABTS assay. The HPLC‐MS analysis identified some phenolic compounds present in the peel extracts such as epigallocatechin gallate, quercetin and resveratrol and additionally to them, rutin for the seed extracts.

## 1. Introduction

Bioactive compounds are chemical molecules that are commonly found in different parts of fruits, vegetables, and grains. They have medicinal properties and can help prevent various diseases such as cancer, diabetes, cardiovascular disease, among others. These substances are considered secondary metabolites and play a defensive role in plants against attacks by microorganisms. These substances have been grouped according to their chemical structure into different groups, which include alkaloids, terpenes, carotenoids, and phenolic compounds [1]. Phenolic compounds are natural substances that originate in the Shikimate/phenylpropanoid metabolic pathway and are characterized by an aromatic ring with one or more hydroxyl substituents. They are further subdivided into phenolic acids, flavonoids, tannins, stilbenes, and lignans [2]. On the other hand, based on the number of phenolic rings and the essential structural elements present in the side chains linked to these rings, phenolic compounds can be subdivided into flavonoids and nonflavonoids [3].

Phenolic compounds are substances of great interest due to the different identified bioactivities, among which can be mentioned as follows: the ability to modify the digestion of macronutrients, prebiotic effects on the intestinal flora, and the reduction of oxidative and inflammatory stress. The reduction of oxidative stress is explained by the antioxidant properties of polyphenols, specifically their ability to capture free radicals and chelate metals [4, 5].

In particular, oxidative stress is linked to a disruption in the balance of pro‐oxidants (free radicals, ROS, and RNS substances) and antioxidants, which is caused by various external and internal factors, including exposure to radiation and pollutants and the generation of by‐products during the body′s various metabolic processes. Oxidative stress has been associated with a wide variety of pathologies such as diabetes, atherosclerosis, cancer, cardiovascular, and neurodegenerative diseases including Parkinson′s and Alzheimer′s, infertility, among others [2, 6, 7].

As mentioned above, bioactive compounds, especially phenolic compounds, can be found in various parts of fruits, vegetables, and grains. Specifically, the *Passiflora* genus includes more than 525 species native mainly to neotropical areas of North, Central, and South America. Among these species, passion fruit or “maracuya amarillo” (*Passiflora edulis flavicarpa*), “gulupa” or “maracuya morado” (*Passiflora edulis Sims fo edulis*), “curuba” or “tumbo” (*Passiflora mollisima*), and “granadilla” (*Passiflora ligularis*) are the most valued on the international market. However, *P. ligularis* and *P. mollissima* are not well known as the others due to climatic restrictions that limit their production [8–10]. Several studies have demonstrated the antioxidant, antimicrobial, and antidiabetic properties of extracts obtained from different parts of plants of the *Passiflora* species. Sasikala et al. [11] investigated the antioxidant potential of extracts obtained from different parts (root, leaves, flower, peel, and seed) of *P. foetida L.* using petroleum ether, ethanol, and water as solvents, with peel extracts showing the best antioxidant characteristics. Saravanan and Parimelazhagan [12] conducted a comparative study of the antioxidant, antimicrobial, and antidiabetic properties of *P. ligularis* pulp extracts obtained with different organic solvents using the percolation method and obtained the highest values of phenolic compound recovery and antioxidant and antidiabetic response in the acetone extracts, concluding that *P. ligularis* pulp could be used as a potential antioxidant and antimicrobial agent. It should be noted that the focus of this study was not on the study of other parts of the fruit, and ethanol was not considered among the solvents. Shanmugam et al. [13] identified 15 polyphenolic compounds in ethanolic extracts of *P. subpeltata* pulp, among which protocatechuic acid, ferulic acid, vanillic acid, epicatechin, p‐coumaric acid, cinnamic acid, eriodictyol, and quercetin‐3‐glucoside stood out. These extracts showed high DPPH+ and ABTS+ cationic radical scavenging activities and *α*‐amylase and *α*‐glucosidase enzyme inhibition capacity, results that could justify their use in the preparation of nutraceutical products to prevent Type II diabetes. Another study conducted on extracts obtained from other species such as *P. ligularis, P. edulis, P. edulis flavicarpa, and P. mollissima*, using pressurized hot water extraction, has identified around 57 phenolic compounds with antioxidant capacity [8].

Studies have shown over time that conventional extraction methods such as Soxhlet, infusion, magnetic stirring, and maceration have been replaced and/or supplemented with solid–liquid extraction methods intensified with other energy sources, including ultrasound, because conventional methods require extensive extraction times and non‐GRAS solvents [14]. Ultrasound‐Assisted Extraction (UAE) uses high‐frequency waves to weaken cell walls and improve mass transfer during extraction, as well as operating at low temperatures, which minimizes thermal degradation of compounds, preserving their bioactivity. Depending on the solvents used, this technique is aligned with green extraction technologies and has been successfully used for the extraction of phenolic compounds.

The objective of this study was to evaluate phenolic content and in vitro antioxidant activity of extracts obtained from different parts of the fruit—pulp, peel, and seeds—of the granadilla fruit (*P. ligularis*) using the UAE method, with ethanol and methanol as solvents.

## 2. Materials and Methods

### 2.1. Raw Material

Ripe granadilla (*P. ligularis*) fruit was obtained from a local producer in the Chichihuaya sector, Quiaca District, Sandia Province, Puno Department, Peru. Fruit ripeness was assessed based on physical characteristics, including color, flavor, size, shape, and texture. The fruit was then weighed, selected, washed, and disinfected with a 4% sodium hypochlorite solution.

### 2.2. Reagents

Folin–Ciocalteu reagent, 2,2 ^′^‐azino‐bis (3‐ethylbenzothiazoline‐6‐sulfonic acid) reagent and Trolox were purchased from Merck Sigma‐Aldrich (United States). The other reagents used were of analytical grade.

### 2.3. Acidity and Total Soluble Solids (TSS) Determination

Ripe *P. ligularis* fruits were selected for TSS and acidity determination. TSS was measured according to AOAC method 932.12, using a digital refractometer calibrated in °Brix. For analysis, 1 mL of pulp was placed in the refractometer, and the result was recorded at room temperature (25°C). Acidity was determined according to the AOAC method 942.15. Five milliliter of pulp was measured, to which 10 mL of distilled water was added. The sample was subsequently titrated with 0.1 N NaOH until the equivalence point was reached, recording the volume of titrant consumed [15].

### 2.4. Proximate Composition

This analysis was performed on each part of the fruit, by evaluating the peel, seeds, and pulp separately. The analyses were performed according to AOAC methods. Total protein content was determined by the Kjeldahl method (AOAC 960.52), using a nitrogen conversion factor of 6.25. Ash quantification was performed by muffle incineration at 550°C (AOAC 923.03). Carbohydrates were determined according to AOAC method 31.043. Moisture content was measured using the oven‐drying method at 105°C (AOAC 925.45). Fat was determined by Soxhlet extraction (AOAC 920.39). Fiber was analyzed using AOAC 991.43. Finally, total dietary fiber was determined according to AOAC method 991.43, considering the sum of soluble and insoluble dietary fiber [15].

### 2.5. Extract Preparation

First, the seeds were pulverized using a commercial blender, whereas the peels were manually cut into pieces approximately 2.5–5 mm in size. Next, the seeds, peels, and pulp were placed in polypropylene bags, sealed, and stored at −20°C until further analysis.

UAE was used to obtain extracts from seed and peel, following the procedure described by Leporini et al. [16] with some modifications. Briefly, 14.4 g of sample was weighed and mixed with 100 mL of ethanol or methanol in a flask. Three consecutive extraction cycles (1 h each) were performed using an ultrasound bath (Daihan Scientific Co., mod. WUC‐D06H, South Korea) at a frequency of 40 kHz and 30°C. After each cycle, the mixture was filtered through Whatman No. 4 filter paper. Subsequently, the solvent was removed using a rotary evaporator (Büchi Labortechnik AG, mod. R‐II, Switzerland) at 40°C, with a vacuum pressure of −20 mmHg [16].

In the case of fresh pulp, no extraction step was performed. Instead, the pulp was centrifuged at 1000 rpm and the supernatant was filtered through Whatman No. 4 filter paper. Finally, the material obtained was stored in polypropylene bags at −20°C prior to analysis.

### 2.6. Total Phenolic Compounds

The quantification of total phenolic compounds was carried out following the method of Singleton and Rossi [17] published in AOAC [15], with some modifications. First, a 20% anhydrous sodium carbonate (Na_2_CO_3_) solution was prepared. For the gallic acid calibration curve, concentrations between 40 and 200 ppm REF were used.

Two hundred fifty microliter of each sample and a blank sample were set aside. A total of 3.75 mL of distilled water, 250 *μ*L of Folin–Ciocalteu reagent, and 750 *μ*L of the 20% sodium carbonate solution were added to each tube. Afterwards, the mixtures were incubated for 120 min away from light.

The absorbance was measured at 765 nm using a spectrophotometer (Biobase Bioland Co, mod. BK‐UV1000, Shandong, China). The results were expressed in gallic acid equivalent (GAE) units, based on the calibration curve obtained under these conditions.

### 2.7. Antioxidant Capacity

#### 2.7.1. Ferric Reducing Antioxidant Power (FRAP)

The method used was the one described by Benzie and Strain [18], with modifications by Ghasemzadeh et al. [19]. Thirty milliliter of the FRAP solution was prepared by mixing 25 mL of acetate buffer (300 mM, pH 3.6), 2.5 mL of a TPTZ solution (10 mM in 40 mM HCl), and 2.5 mL of a ferric chloride hexahydrate solution (FeCl_3_·6H_2_O, 20 mM). To 2850 *μ*L of this solution, 150 *μ*L of the sample extract was added, and the mixture was incubated for 30 min in the dark. Subsequently, the absorbance was measured at 593 nm using a spectrophotometer (Biobase Bioland Co., mod. BK‐UV1000, Shandong, China). For the standard curve, reference solutions of Trolox, gallic acid, and ferric sulfate were used at previously determined concentrations, allowing the results to be expressed in equivalents of these antioxidant substances.

#### 2.7.2. ABTS+ Cationic Radical Scavenging Activity

The antioxidant capacity was determined using the ABTS radical cation decolorization assay according to Re et al. [20] with slight modifications. The ABTS•+radical cation was generated by reacting 7 mM ABTS solution with 2.45 mM potassium persulfate and allowing the mixture to stand in the dark at room temperature for 16–24 h before use. The resulting solution was diluted with phosphate buffered saline (PBS) (pH 7.4; 10 mM; 150 mM NaCl) until an absorbance of 0.70 ± 0.02 at 734 nm was obtained. An aliquot of the extract (100 *μ*L) was mixed with the diluted ABTS•+solution (10 mL), and the reaction mixture was incubated at room temperature for 6 min. The decrease in absorbance was measured at 734 nm using a UV–Vis spectrophotometer against a reagent blank. Calibration curves were obtained using Trolox solutions that were prepared with the different solvents used in this work, and the antioxidant activity was quantified as Trolox equivalent antioxidant capacity (TEAC). The results were expressed as *μ*mol Trolox equivalents (TE) per 100 mL of extract and per 100 g of dry extract.

### 2.8. Liquid Chromatography Coupled to Mass Spectrometry (LC–MS) Analysis of Phenolic Compounds

The analysis of phenolic compounds was performed using LC–MS. Chromatographic separations were carried out on a Nexera XSi liquid chromatograph equipped with high‐pressure mixing pumps and a Shim‐pack C18‐AQ HP column (1.9 *μ*m, 2.1 × 100 mm). The column was maintained at 40°C, and elution was performed under a gradient program using acetonitrile containing 0.1% formic acid and water (5/95, *v*/*v*), at a constant flow rate of 0.4 mL/min. The chromatographic system was interfaced with a single‐quadrupole LCMS‐20250 detector operating under DUIS ionization. Instrumental parameters included a nebulization gas flow of 2 L/min and desolvation and interface temperatures of 400°C and 250°C, respectively. A set of nine phenolic standards (ferulic acid, resveratrol, epigallocatechin, p‐coumaric acid, epicatechin, quercetin, rutin, chlorogenic acid, and kaempferol) was used for qualitative confirmation and quantitative evaluation. Standards were analyzed in selected ion monitoring (SIM) mode. Compound identification was established by matching retention times and ion‐trace profiles between standards and sample extracts, ensuring analytical selectivity. Before injection, all extracts were diluted in acetonitrile/water (50/50, *v*/*v*), filtered through 0.22 *μ*m PVDF membranes, and injected at 2 *μ*L [21].

## 3. Results and Discussion

### 3.1. Physicochemical Analysis

The titratable acidity of the analyzed sample was 0.4036 g of citric acid per 100 g of sample, whereas the TSS content reached 16.5° Brix. In contrast, de Souza Silva et al. [22] reported a titratable acidity of 3.36 g of citric acid per 100 g and a soluble solids content of 10.8° Brix for ripe *Passiflora cincinnata* pulp. When comparing these values with those obtained in the present study, it can be seen that both acidity and soluble solids were higher in the sample from the present study.

The differences in titratable acidity and soluble solids concentration of the fruit can be attributed to various factors, such as variety or species, ripeness, and agroclimatic conditions of the growing region [23]. These variables can influence the metabolism of carbohydrates and organic acids, which would explain the variations observed in acidity and soluble solids content.

### 3.2. Proximal Composition

The proximal analysis of the granadilla (*P. ligularis)* revealed differences in the composition of the different parts of the fruit (peel, pulp, and seed). The results, presented in Table 1, show that the seed is the fraction with the highest protein (26.33 g/100 g d.b.) and fat (23.77 g/100 g d.b.) content, whereas the peel is distinguished by its high fiber content (38.00 g/100 g d.b.). The pulp had the highest moisture content (84.06%) and a high percentage of carbohydrates (90.99% w.b.).

**Table 1:** Proximal composition (%) of *Passiflora ligularis* of fresh sample on a dry basis (d.b.) and wet basis (w.b.).

Description	*Passiflora*	Peel		Juice/pulp		Seed		References
		w.b.	d.b.	w.b.	d.b.	w.b.	d.b.	
Moisture	*P. ligularis*	77.84 ± 0.04^b^	—	84.06 ± 0.09^a^	—	49.79 ± 0.08^c^	—	
	*P. edulis f. edulis*	87				46		24
	*P. caerulea*	94.25 ± 0.26						28
	*P. edulis Sims*			72.93				25
	*P. cincinnata*			83.45 ± 0.29				26
	*P. cincinnata*			90.02 ± 0.11				27
Ashes	*P. ligularis*	1.38 ± 0.03^b^	6.23 ± 0.12^a^	0.74 ± 0.02^c^	4.64 ± 0.13^b^	1.49 ± 0.02^a^	2.97 ± 0.04^c^	
	*P. edulis f. edulis*		7.93	1.02–1.29			1.34–1.85	24
	*P. edulis Sims*			0.8				25
	*P. cincinnata*			1.47 ± 0.07				26
	*P. cincinnata*			0.08 ± 0.09				27
	*P. caerulea*		13.29 ± 0.04					28
Fat	*P. ligularis*	0.15 ± 0.02^b^	0.68 ± 0.09^b^	0.1 ± 0.03^b^	0.63 ± 0.17^b^	11.94 ± 0.04^a^	23.77 ± 0.08^a^	
	*P. edulis f. edulis*		0.4–4.89	4.8–7.0			14.9–30.1	24
	*P. edulis Sims*			0.7				25
	*P. cincinnata*			3.83 ± 0.30				27
	*P. caerulea*		10.25 ± 0.12					28
Proteín (X/6.25)	*P. ligularis*	0.66 ± 0.01^b^	2.98 ± 0.05^b^	0.43 ± 0.03^b^	2.70 ± 0.22^b^	13.22 ± 0.01^a^	26.33 ± 0.02^a^	
	*P. edulis f. edulis*		6.47–7.50				12.2–13.2	24
	*P. caerulea*		11.6 ± 0.44					28
	*P. edulis Sims*			2.2				25
	*P. cincinnata*			0.84 ± 0.06				27
Fiber	*P. ligularis*	8.42 ± 0.05^b^	38 ± 0.24^a^	0.17 ± 0.03^c^	1.09 ± 0.16^c^	14.80 ± 0.07^a^	29.48 ± 0.13^b^	
	*P. edulis f. edulis*		61.7				43.8–55.1	24
	*P. caerulea*		62.14 ± 2.62					28
Carbohydrates	*P. ligularis*	11.55 ± 0.08^b^	52.12 ± 0.38^b^	14.50 ± 0.03^a^	90.99 ± 0.16^a^	8.76 ± 0.07^c^	17.45 ± 0.14^c^	
	*P. edulis f. edulis*		80.7	5.54–23.4			69.9	24
	*P. caerulea*		64.86 ± 0.00					28
	*P. edulis Sims*			23.38				25
	*P. cincinnata*			5.53 ± 0.64				27
Energy ^*^		224.98	302.48	67.03	382.96	60.96	448.08	

*Note:* The asterisk (∗) denotes energy expressed in (kcal/100 g). Values are expressed as mean ± standard deviation (*n* = 3). Different superscript letters within the same row indicate statistically significant differences (*p* < 0.05) according to one‐way ANOVA and Tukey′s multiple comparison test (95% confidence level).

Seeds of *P. ligularis* showed a moisture content of 49.79%, higher than the 46% reported for *Passiflora edulis f. edulis* [24]. The fiber content was 29.48% (d.b.), lower than the reported range of 43.8%–55.1% (d.b.) for *P. edulis f. edulis* [24]. The fat content in *P. ligularis* seeds was 23.77% (d.b.), within the range of 14.9%–30.1% (d.b.) reported for *P. edulis f. edulis*, whereas the protein content (26.33% d.b.) was markedly higher than the 12.2%–13.2% (d.b.) reported for *P. edulis f. edulis* [24]. The ash content of *P. ligularis* seeds was 2.97% (d.b.), exceeding the 1.34%–1.85% (d.b.) range reported for *P. edulis f. edulis*, whereas the carbohydrate content (17.45% d.b.) was considerably lower than the 69.9% (d.b.) reported for this species [24]. Altogether, these results indicate that *P. ligularis* seeds are relatively richer in protein, fat, and minerals, and poorer in carbohydrates and fiber, compared with *P. edulis f. edulis* [25].

The moisture content of *P. ligularis* pulp was 84.06%, higher than the 72.93% reported for *P. edulis Sims* [26] and similar to the 83.45% reported for *P. cincinnata* [27], and lower than the 90.02% reported for *P. cincinnata* [28]. This value lies within the 72%–86% range documented for *P. edulis f. edulis* pulp [24]. The carbohydrate content of *P. ligularis* pulp was 14.50% (w.b.), within the range of 5.54%–23.4% (w.b.) reported for *P. edulis f. edulis* [24], lower than the 23.38% (w.b.) reported for *P. edulis Sims* [26] and higher than the 5.53% (w.b.) reported for *P. cincinnata* [28]. The fat content in *P. ligularis* pulp was 0.10% (w.b.), lower than the values reported for *P. edulis Sims, P. cincinnata*, and *P. edulis f. edulis* [24, 26, 28]. The protein content was 0.43% (w.b.), lower than the 2.2% (w.b.) reported for *P. edulis Sim* [26], but higher than the 0.083% (w.b.) reported for *P. cincinnata* [28]. The ash content in *P. ligularis* pulp was 0.74% (w.b.), comparable to the 0.8% (w.b.) reported for *P. edulis Sims* lower than the 1.47% (w.b.) reported for *P. cincinnata* [27], higher than the 0.08% (w.b.), reported for *P. cincinnata* [28], and below the 1.02–1.29% (w.b.) range described for *P. edulis f. edulis* [24]. These results show that *P. ligularis* pulp presents typical moisture levels for passion fruit pulps and an intermediate contribution of carbohydrates, protein and minerals compared with related species.

The peel of *P. ligularis* showed a moisture content of 77.84%, lower than the 87% and 94.25% reported for *P. edulis f. edulis* and *P. caerulea*, respectively. Its carbohydrate content was 52.12% (d.b.), lower than the 80.7% and 64.86% (d.b.) reported for *P. edulis f. edulis* and *P. caerulea*, respectively [24, 29]. The fiber content of *P. ligularis* peel was 38% (d.b.), lower than the 61.7% (d.b.) and 62.14% (d.b.) reported for *P. edulis f. edulis* and *P. caerulea*. The ash content was 6.23% (d.b.), lower than the 7.93% (d.b.) and 13.29% (d.b.) reported for *P. edulis f. edulis* and *P. caerulea*, respectively [24, 29]. The protein content of *P. ligularis* peel was 2.98% (d.b.), lower than the 6.47%–7.50% (d.b.) and 11.60% (d.b.) reported for *P. edulis f. edulis* and *P. caerulea*, respectively [24, 29], whereas the fat content (0.68% d.b.) was within the 0.4%–4.89% (d.b.) range reported for *P. edulis f. edulis* [24] and lower than the 10.25% (d.b.) reported for *P. caerulea* [29]. Thus, although *P. ligularis* peel still contains substantial fiber and minerals, it is generally less concentrated in nutrients than the peels of *P. edulis f. edulis* and *P. caerulea* [25].

This differentiated distribution of nutrients supports the potential use of seeds as a source of protein and oil, pulp as a moisture and carbohydrate‐rich matrix for beverages and pulped products, and peel as a fiber and mineral‐rich fraction for the development of functional ingredients and other value‐added products in the food sector.

### 3.3. Total Phenolic Content (TPC)

Table 2 shows the results for total phenolic compound content. Of all the extracts analyzed, the methanolic seed extract had the highest TPC, reaching a value of 36.26 mg GAE/g dry extract, which is almost four times higher than the ethanol seed extract (9.25 mg GAE/g dry extract). In the case of extracts obtained from the peel, the methanolic extract showed a TPC content of 1.51 mg GAE/g dry extract, a concentration that was almost twice as high as that obtained in the ethanol extract (0.80 mg GAE/g dry extract). In this sense, there is clear evidence of the influence of the type of solvent on the extraction of phenolic compounds, with methanol being more efficient. In the case of fresh pulp, the TPC content was lower (0.35 mg GAE/g pulp) than that extracted with solvents. This behavior could be explained by the findings of Lezoul et al. [30], who indicate that methanol has a higher polarity than ethanol, which is why highly polar phenolic acids, such as cinnamic or benzoic acid, can be extracted more easily, leading to an overall increase in the polyphenol content of the extracts.

**Table 2:** Total phenolic content in pulp and extracts of *Passiflora ligularis.*

Solvents	Extracts	TPC
		mg GAE/g dry extract
	Pulp ^∗^	0.35 ± 0.01^e^
Ethanol	Peel	0.80 ± 0.00^d^
	Seeds	9.25 ± 0.01^b^
Methanol	Peel	1.51 ± 0.01^c^
	Seeds	36.26 ± 0.20^a^

*Note:* The asterisk ( ^∗^) denotes fresh pulp without solvents. Values are expressed as mean ± standard deviation (*n* = 3). Different superscript letters indicate statistically significant differences (*p* < 0.05) according to one‐way ANOVA followed by Tukey′s multiple comparison test at a 95% confidence level.

Abbreviation: TPC, total phenolic compound content.

Dos Reis et al. [29] characterized three species of *Passiflora*: yellow passion fruit (*Passiflora edulis Sims fo. flavicarpa*), purple (*Passiflora edulis Sims fo. edulis*), and orange passion fruit (*Passiflora caerulea*), finding TPC values of 346.69, 325.00, and 429.33 mg GAE/100 g dry extract, respectively, for ethanolic extracts obtained from the seeds of these species.

On the other hand, several investigations have been found which have studied the content of phenolic compounds in the peels of various species of the genus *Passiflora*. Gamarra‐Castillo et al. [31] reported a TPC value of 1904.45 mg GAE/100 g dry extract for *P. edulis f. edulis* obtained with an acidified methanol solution (50:50 v/v, 0.8% hydrochloric acid) and an aqueous acetone solution (80:20 v/v). This value is approximated to the value obtained by Dos Reis et al. [29], who found values of 1061.87, 1570.80, and 2584.91 mg GAE/100 g dry ethanolic extracts of yellow, purple, and orange passion fruit peels. Other studies show different results due to the use of different extraction methods and solvents.

Hot water pressurized extracts of *P. ligularis* showed a content of 5.08 mg GAE/g dry extract and for *P. Mollisima* a content of 30.9 mg GAE/g dry extract. The 70% ethanolic extract of *P. Edulis* had a TPC of 32.6 mg GAE/g dry extract. These variations are likely due to various conditions in the extract obtaining process, such as time, temperature, sample extraction volumes, as well as the nature of the food matrix and the polarity of the solvent [8, 32, 33].

In the case of fresh pulp, a TPC value of 0.35 mg GAE/g pulp (35 mg GAE/100 g pulp) was obtained, which is significantly lower when compared with peel and seed extracts. Similar values were obtained by Rotta et al. [34], who worked with pulp from *P. edulis* (≈41 mg GAE/100 g pulp), *P. alata* (≈43 mg GAE/100 g pulp), and *P. ligularis* (≈20 mg GAE/100 g pulp). Carmona‐Hernandez et al. [33] reported values of 40.50, 47.12, and 46.84 mg GAE/100 g pulp for freeze‐dried pulp extracts of *P. edulis Flavicarpa*, *P. edulis Sims*, and *P. ligularis Juss*, respectively. These extracts were processed using the solid‐liquid extraction method and 80% ethanol as the solvent. Considering that, for the present study, the pulp was not subjected to any extraction process or contact with solvents, the results obtained are consistent with those reported by the authors. However, Santos et al. [28] determined TPCs in unpasteurized *P. cincinnata* pulp, obtaining a value of 3.12 mg GAE/100 g pulp, which is significantly lower than that obtained in the present study.

Other studies have reported different TPC values for pulp extracts from *Passiflora* species, in which different solvents were used and different extraction processes were applied, making comparison difficult. Shanmugam et al. [35] reported an TPC of 691.90 GAE mg/g pulp of *P. leschenaultii* and 172 GAE mg/g dry methanol extract. Carmona‐Hernandez et al. [33] obtained values of 79.21 and 54.22 mg GAE/100 g of pulp *P. ligularis* fruit using extraction with 70% acetone and methanol solution, respectively. Guevara et al. [36] reported a value of 324.18 mg/100 g pulp for *P. quadrangularis* pulp after freeze‐drying and extraction with a hydroalcoholic solution. Finally, Saravanan and Parimelazhagan [12] used petroleum ether (102.40 mg GAE/g dry extract), chloroform (125.70 mg GAE/g dry extract), methanol (137.90 mg GAE/g dry extract), and acetone (640.70 mg GAE/g dry extract), with the latter being the one with the best performance.

### 3.4. Antioxidant Activity

#### 3.4.1. FRAP Assay

In the case of antioxidant capacity measured by the FRAP method, significant differences can be observed between the results of the different samples analyzed (Table 3). The highest FRA*P* value was recorded for the methanolic extract obtained from the seeds (2669.357 *μ*mol TE/g dry extract), which was 1. Seven times higher than that recorded for the ethanolic extract (1528.134 *μ*mol TE/g dry extract), which corresponds to the results of TPCs in which methanol was more efficient for the extraction of these compounds. In the case of extracts obtained from the peel, the same behavior can be observed and, as expected, the lowest values recorded were in the pulp since it was not subjected to any extraction process.

**Table 3:** Antioxidant activity results for extracts and fresh pulp of *Passiflora ligularis.*

Solvent	Sample	FRAP	
		*μ*mol TE/g dry extract	*μ*mol Fe (II)/g dry extract
	Pulp	5.634 ± 0.053^e^	10.004 ± 0.101^e^
Ethanol	Peel	131.431 ± 1.061^d^	239.064 ± 2.031^d^
	Seed	1528.134 ± 27.551^b^	2740.884 ± 52.768^b^
Methanol	Peel	397.135 ± 5.626^c^	1162.474 ± 16.399^c^
	Seed	2669.357 ± 51.324^a^	7816.902 ± 149.591^a^

*Note:* Values are expressed as mean standard deviation (*n* = 3). Ethanol (EtOH) at 99.5%, methanol (MeOH). Results are shown in *μ*mol equivalent of Trolox (TE) and FeSO4.H2O (Fe[II]) to facilitate comparisons with results from other authors. Values are expressed as mean ± standard deviation (*n* = 3). Different superscript letters within each column indicate statistically significant differences (*p* < 0.05) according to one‐way ANOVA followed by Tukey′s post hoc test.

The pulp of *P. ligularis* reached a FRA*P* value of 10.00 *μ*mol Fe(II)/g dry extract. Previous studies have reported different antioxidant capacity values for pulps from different *Passiflora* varieties. Saravanan and Parimelazhagan [12] reported FRAP values between 20.51 and 43.06 mmol Fe(II)/mg dry extract for *P. ligularis* pulp extracts using various organic solvents and using the percolation method. Loizzo et al. [37] found different FRAP values for ethanolic extracts of *P. pinnatistipula* pulp (3.2 *μ*mol Fe(II)/g dry extract), *P. tripartita* (64.1 *μ*mol Fe(II)/g dry extract), and for an extract of pulp and seeds of *P. ligularis* (42.9 *μ*mol Fe(II)/g dry extract). Guevara et al. [36] determined a FRAP reducing capacity of 387.89 *μ*mol Trolox/g of fresh fruit for hydroalcoholic extracts of lyophilized *P. quadrangularis* pulp. Borgonovi et al. [38] obtained a FRAP value of 2.87 *μ*mol TE/g sample for lyophilized pulp extract of *P. edulis f. flavicarpa Deg*. obtained with a methanol and acetone solution (70% methanol + 50% acetone [v/v, 1:1]). The notable differences between the values can be explained by the diversity of *Passiflora* varieties, the different methodologies used for sample treatment, the extraction process, the type of solvents, and the calculation basis for reporting the results.

The FRAP antioxidant capacity for the methanolic extract of *P. ligularis* peel was significantly higher (1162.474 *μ*mol Fe(II)/g dry extract.) compared with the ethanolic extract (239.064 *μ*mol Fe(II)/g dry extract), indicating that the compounds responsible for conferring ferric ion reducing capacity showed greater affinity for methanol than for ethanol, a response that is consistent with the behavior observed in the determination of total phenolic compounds. Loizzo et al. [37] obtained FRA*P* values of 40.5, 22.7, and 39.9 *μ*mol Fe(II)/g dry ethanolic extracts for ethanolic extracts obtained by cold extraction from the peels of *P. pinnatistipula, P. tripartita*, and *P. ligularis*, respectively. Herrera‐Ramirez et al. [39] worked with purple passion fruit (*P. edulis f. edulis Sims*) peel, which was lyophilized, ground, and subsequently subjected to extraction with 96% ethanol acidified with citric acid, obtaining a FRAP value of 5.29 *μ*mol Trolox/g dry extract. Apparently, ultrasound extraction improved the extraction yield of the components responsible for the FRAP response. However, a direct comparison it is not possible due to the difference in the concentration of solids in the reference extracts.

In the case of seeds, the same solvent affinity behavior was observed, that is, the methanolic extract (7816.902 *μ*mol Fe(II)/g dry extract) showed greater antioxidant activity than the ethanolic extract (2740.884 *μ*mol Fe(II)/g dry extract). Loizzo et al. [37] reported activities of 28.7 and 92.0 *μ*mol Fe(II)/g dry ethanolic extracts from *P. pinnatistipula* and *P. tripartita* seeds, respectively, and 42.9 *μ*mol Fe(II)/g for a mixture of pulp and seed dry extracts from *P. ligularis*, all of which were processed by cold extraction. Apparently, the results obtained in this study were much higher than those reported in the literature, which could be attributed to the effect of ultrasound waves. However, it is not possible to make a real comparison since we do not know the exact concentration of solids present in the extracts obtained by other authors.

During UAE, ultrasonic mechanical waves, which have the ability to propagate in elastic media such as solvents, generate the implosion of cavitational bubbles, which in turn produce microjets with extreme pressure and temperature conditions that impact cell membranes. This causes them to rupture and consequently increases porosity, which accelerates the penetration of the solvent into the biological material and facilitates the release of the bioactive compounds contained therein [40, 41].

#### 3.4.2. Antioxidant Capacity by ABTS^+0^ Cationic Radical Scavenging

The antioxidant capacity results obtained using the ABTS method are presented in Table 4. As observed in the results for TPC and FRAP, the best responses on antioxidant activity were achieved in methanolic extracts. The highest antioxidant activity was exhibited by the methanol extracts of seeds (1161.507 mmol TE/100 g extract) and peels (232.373 mmol TE/100 g extract), which were 1.54 and 1.04 times higher than the ethanol extracts (754.077 mmol TE/100 g seed extract and 221.569 mmol TE/100 g peel extract).

**Table 4:** ABTS assay of pulp and peel extracts, seeds of *Passiflora ligularis.*

Solvent	Sample	ABTS	
		*μ*mol TE/100 mL	mmol TE/100 g
Methanol	Seed	51454.762 ± 4003.154^b^	1161.507 ± 90.365^d^
	Peel	7296.508 ± 114.376^a^	232.373 ± 3.643^b^
Ethanol	Seed	46677.363 ± 3160.404^b^	754.077 ± 51.057^c^
	Peel	4874.525 ± 185.906^a^	221.569 ± 8.45^b^
Pulp		922.335 ± 51.498^a^	5.239 ± 0.293^a^

*Note:* Values are expressed as mean ± standard deviation (*n* = 2). Ethanol (EtOH) at 99.5%, methanol (MeOH). Results are shown in *μ*mol Trolox equivalent (TE). Different superscript letters within each column indicate statistically significant differences (*p* < 0.05) according to one‐way ANOVA followed by Tukey′s post hoc test.

The lowest antioxidant response was obtained in the pulp sample with a value of 5.239 mmol TE/100 g dry pulp.

Lezoul et al. [30] observed the same behavior when evaluating different solvents (methanol, ethanol, and acetone) for the extraction of bioactive compounds from inedible parts of *P. caerulea L.* using maceration and decoction extraction methods. The highest values for phenolic compound extraction and the corresponding antioxidant capacity were obtained in the methanolic extracts, regardless of the extraction method used, associating the response with the higher polarity exhibited by methanol compared with other solvents such as ethanol. This characteristic facilitates the extraction of polar phenolic compounds such as phenolic acids, which was also verified by the increase in the concentration of total phenolic compounds when the concentration of methanol was increased in the extraction process.

The pulp of *P. ligularis* exhibited the lowest antioxidant activity value (5.239 mmol TE/100 g dry pulp). Rotta et al. [34] reported ABTS values of 575 *μ*mol TE/100 g extract for *P. ligularis* and 550 *μ*mol TE/100 g extract for *P. alata* using the QuEChERS extraction method. Shanmugam et al. [35] found strong antioxidant capacity (9760.44 *μ*mol TE/100 g extract) for freeze‐dried *P. leschenaultii* pulp, although this value decreased considerably when extraction was performed with methanol (1323.89 *μ*mol TE/g extract). On the other hand, the extraction of *P. ligularis* pulp with acetone yielded an ABTS value of 9800.94 *μ*mol/L TE/100 g of extract [34]. Finally, Loizzo et al. [37] reported ABTS values, in terms of IC_50_, for ethanolic extracts of *P. pinnatisipula* (151.7 *μ*g/mL) and *P. tripartita* (50.1 *μ*g/mL) pulp.

In the case of *P. ligularis* peel extracts, ABTS values of 221.569 mmol TE/100 g dry extract and 232.373 mmol TE/100 g dry extract were recorded for the ethanolic and methanolic extracts, respectively. These values were higher than those found by Domínguez‐Rodríguez et al. [8], who worked with different *Passiflora* species such as *P. ligularis* (5.0 mmol TE/100 g dry extract), *P. edulis* (201.0 mmol TE/100 g dry extract), *P. edulis flavicarpa* (8 mmol TE/100 g dry extract), and *P. mollisima* (224 mmol TE/100 g dry extract). It should be noted that these extracts were obtained by the pressurized hot water extraction method using a mixture of water/ethanol/formic acid (94:5:1, vol%) as the solvent. On the other hand, Dos Reis et al. [29] reported ABTS values expressed in IC_50_ for ethanolic extracts of the peel of *P. edulis Sims fo. Flavicarpa* (2.22 g/100 mL), *P*. *edulis Sims fo. Edulis* (9.37 g/100 mL), and *P. caerulea* (2.95 g/100 mL).

The seed extracts showed significantly higher values compared with the peel extracts and fresh pulp (754.077 mmol TE/100 g dry extract using ethanol and 1161.507 mmol TE/100 g dry extract using methanol), with methanol being the most efficient solvent for the extraction of compounds with ABTS cationic radical scavenging capacity. Dos Reis et al. [29] obtained ABTS values expressed in IC_50_ for ethanolic extracts of the peel of *P. edulis Sims fo. Flavicarpa* (3.84 g/100 mL), *P. edulis Sims fo. Edulis* (4.76 g/100 mL), and *P. caerulea* (3.87 g/100 mL).

### 3.5. Identification of Phenolic Compounds

Analysis of the pulp, seeds, and peel of *P. ligularis* showed that the levels of epigallocatechin gallate (EGCG) and quercetin were significantly higher in the peel than in the other fractions.

In terms of microgram per gram of dry extract (Table 5), the methanolic extracts of peel reached 93.5 *μ*g/g of EGCG and 12.88 *μ*g/g of quercetin, whereas with ethanol, 43.2 and 5.60 *μ*g/g, respectively, were obtained. In comparison, the seeds showed intermediate values (2.16–4.18 *μ*g/g of EGCG and 2.28 *μ*g/g of quercetin), and the pulp contributed only 0.84 and 0.71 *μ*g/g. When expressed as dry matter (d.m.) (Table 6), these values correspond to approximately 1.79–1.83 mg of EGCG and 0.23–0.25 mg of quercetin/100 g of peel, compared with 0.43 mg and 0.36 mg in the pulp and 0.13–0.14 mg and 0.07–0.14 mg in the seeds. Rutin and resveratrol were mainly detected in the seeds: the former in a range of 0.40–0.79 *μ*g/g (0.025 mg/100 g), and the latter between 0.70 and 1.48 *μ*g/g (0.044–0.047 mg/100 g). The pulp contained traces of rutin (0.16 *μ*g/g; 0.079 mg/100 g), and no resveratrol was detected, whereas the peel only showed resveratrol in low concentrations (0.811 *μ*g/g, equivalent to 0.034 mg/100 g) and no rutin. These results confirm that phenolic compounds tend to be concentrated in by‐products (peel and seeds) rather than in the edible pulp. It should be noted that the presence of cyanidin‐3‐glucoside, chlorogenic acid, epicatechin, p‐coumaric acid, and ferulic acid was also evaluated, although in all cases the values were nondetectable (N.D.).

**Table 5:** Concentration (*μ*g/g dry extract) of epigallocatechin gallate, quercetin, rutin, and resveratrol in extracts of pulp, seeds, and peel of *Passiflora ligularis* obtained with ethanol and methanol.

Polyphenols	Pulp ^∗^	Seeds in ethanol	Seeds in methanol	Peel in ethanol	Peel/methanol
Epigallocatechin gallate	0.843 ± 0.006^e^	2.161 ± 0.011^d^	4.183 ± 0.012^c^	43.226 ± 0.4^b^	93.547 ± 0.482^a^
Quercetin	0.709 ± 0.111^d^	2.282 ± 0.094^c^	2.289 ± 0.458^c^	5.604 ± 0.027^b^	12.88 ± 0.453^a^
Rutin	0.155 ± 0.002^c^	0.402 ± 0.023^b^	0.788 ± 0.046^a^	N.D.	N.D.
Resveratrol	N.D.	0.7 ± 0.008^b^	1.483 ± 0.033^a^	0.811 ± 0.08^b^	N.D.

*Note:* Values are expressed as mean ± standard deviation (*n* = 3). Different superscript letters within each row indicate statistically significant differences in polyphenol concentration among extract types (*p* < 0.05), according to one‐way ANOVA followed by Tukey′s post hoc test. The asterisk (∗) denotes fresh pulp without solvents.

Abbreviation: N.D., not detected.

**Table 6:** Content (mg/100 g) of epigallocatechin gallate, quercetin, rutin, and resveratrol in the pulp, seeds, and peel of *Passiflora ligularis.*

Polyphenols	Pulp	Seeds in ethanol	Seeds in methanol	Peel in ethanol	Peel/methanol
Epigallocatechin gallate	0.431 ± 0.003^c^	0.136 ± 0.001^d^	0.134 ± 0^d^	1.795 ± 0.017^b^	1.832 ± 0.009^a^
Quercetin	0.363 ± 0.057^a^	0.143 ± 0.006^cd^	0.073 ± 0.015^d^	0.233 ± 0.001^bc^	0.252 ± 0.009^b^
Rutin	0.079 ± 0.001^a^	0.025 ± 0.001^b^	0.025 ± 0.001^b^	N.D.	N.D.
Resveratrol	N.D.	0.044 ± 0^a^	0.047 ± 0.001^a^	0.034 ± 0.003^b^	N.D.

*Note:* Values are expressed as mean ± standard deviation (*n* = 3). Different superscript letters within each row indicate statistically significant differences among extract types (*p* < 0.05), according to one‐way ANOVA followed by Tukey′s post hoc test. Samples sharing the same letter are not significantly different.

Abbreviation: N.D., not detected.

When these results are compared with the literature, it can be seen that the concentrations in *P. ligularis* are modest compared with other food sources. High‐quality green teas contain between 20 and 35 mg of EGCG/g of dry leaves, and some fruits such as Fuji apples reach 1.93 mg/100 g edible portion [42], whereas passion fruit peels provide about 1.8 mg/100 g. In the case of quercetin, foods such as raw capers can contain 233 mg/100 g, and vegetables such as radish leaves or arugula exceed 60 mg/100 g [43], values well above the 0.23–0.25 mg/100 g detected in the peel of *P. ligularis*. A similar pattern was observed for rutin, which is widely present in Tartary buckwheat (6–38 mg/g) [44], but limited to 0.025 mg/100 g in passion fruit seeds. The resveratrol found in seeds (~0.047 mg/100 g) is comparable to that found in roasted peanuts (0.006–0.08 mg/100 g), although much lower than that found in grape skins, which contain 50–100 *μ*g/g (equivalent to 5–10 mg/100 g) [45, 46]. The interspecific differences are also notable: in *P. edulis*, it has been reported that the peel can contain up to 2585 mg of total phenolics and more than 800 mg/100 g of quercetin in the orange variety, values much higher than those observed in *P. ligularis*. In addition, it has been reported that the genus *Passiflora* includes flavonoids such as vitexin, isovitexin, orientin, and isoorientin in proportions of 0.3%–2.85% of total flavonoids, suggesting that passion fruit has a broader spectrum of compounds than those quantified in this study. Finally, in previous studies with *P. ligularis* extracts prepared with 70% methanol, EGCG and epigallocatechin concentrations below 0.1 *μ*g/mL [47], therefore the values obtained in this study (up to 93.5 *μ*g/g of EGCG in the peel) highlight the influence of the matrix and the solvent on the recovery of polyphenols. Taken together, these findings show that *P. ligularis* by‐products, although not an outstanding source of EGCG, quercetin, rutin, or resveratrol in absolute terms, can be considered as potential functional ingredients for enriching foods or supplements, while highlighting the interspecific variability in the *Passiflora* genus and the importance of optimizing extraction processes to maximize the yield of bioactive compounds.

## 4. Conclusion

*P. ligularis*, particularly its seeds, are rich in phenolic compounds with strong antioxidant capacity. UAE with methanol was the most effective treatment yielding extracts with the highest TPC and antioxidant activity (FRAP and ABTS) values. LC–MS analysis confirmed the presence of key phenolic compounds such as EGCG, quercetin, rutin, and resveratrol, mainly in seeds and peels, highlighting the potential of granadilla by‐products as promising sources of antioxidant compounds for food and nutraceutical applications.

## Author Contributions

**Fabiola N. Cuyo-Jallasi:** conceptualization, data curation, funding acquisition, investigation, methodology, project administration, resources, writing—original draft, and writing—review and editing. **Julio C. Rodríguez-Díaz:** supervision, formal analysis, and writing—review and editing. **Harry R. Yucra-Condori:** formal analysis, methodology, and writing—review and editing. **Miriam D. Hubinger:** supervision, validation, and visualization.

## Funding

This work was funded by the Universidad Nacional de San Agustín de Arequipa, Peru (Contract Number TP IB‐50‐2021‐UNSA).

## Disclosure

All authors have read and approved the final manuscript.

## Conflicts of Interest

The authors declare no conflicts of interest.

## Data Availability

The data that support the findings of this study are available from the corresponding author upon reasonable request.
